# The Medical Profession and the Army

**Published:** 1916-04-01

**Authors:** 


### The Hospital

Workers' Newspaper of Administrative Medicine and Institutional Life, Administration, National Insurance and Health.

No. 1555, Vol. LX. SATURDAY, APRIL 1, 1916. PRICE ONE PENNY

230056

### THE MEDICAL PROFESSION AND THE ARMY.

It is impossible to avoid a feeling of sincere sympathy for members of the medical profession who fall within the military age and who are therefore called upon to say whether they are or are not willing to take service in the Army should their services be required. The great majority of these are men who have been in independent practice for a longer or shorter period. They have incurred obligations, both domestic and financial. Toward these obligations the Army pay will be but an inadequate contribution. And a prolonged absence from practice must mean necessarily the loss of patients and a depreciated income. Such, in bald terms, is the position which a large number of medical practitioners are at this moment called upon to face. On the other hand is the certainty that the Army which stands between the country and national disaster is in clamant need of more doctors. This necessity, it is true, has been challenged, but hardly by any responsible authority. It now stands without possibility of contradiction, for we have the plain announcement of the Director-General that even to meet the wastage of war in France he needs every month at least 150 new medical officers. There are losses by death, by wounds, and by sickness, and some officers who have fulfilled their engagement of a year's service are compelled by the exigencies of practice to return to their homes. In addition, it has to be remembered that by the summoning to the Colours of the "Groups" and the "Classes," the Army is constantly being increased in size, and soldiers in training camps, like to those in the trenches, must needs have medical help and oversight. It is not necessary to say to the medical profession that as a matter of imperative patriotic responsibility the situation so depicted must be fully met. The profession has indeed already done great things and made great sacrifices. But the measure of the moment is fixed solely by the standard of sufficiency. Everywhere in the national emergency the demand is not for "much" but for "enough." And there is now written in plain characters the message that whatever personal sacrifices may be involved more doctors must be recruited for the Army.

The necessity being beyond dispute, there remains only the question—How can it be met? Manifestly, one method would be the issue of an appeal for volunteers comparable to those to which recent months have made us accustomed. In substance this method has been in operation since the commencement of the war, and the response to it is seen in the multitudes of civil practitioners now serving as officers in the R.A.M.C. Doubtless a further call direct from the War Office to the profession would bring even yet a number of volunteers. But it is necessary to observe that the situation to-day is very different from that which existed in the early months of the war. Then there were engaged in various forms of hospital and civil practice large numbers of young men who had few responsibilities and to whom new experiences and adventurous undertakings made a ready appeal. Now these, and many others who could no longer claim the advantages of youth, are already serving their King and country. The appeal for further help therefore comes to men whose plan of life is settled, who have given hostages to fortune, and who cannot answer the call except at the risk of interests which both to themselves and to their families are of the first importance. When they answer, as they will answer, it will be not with light hearts keen for enterprise, but under the sense of a deep patriotic obligation and with the anxious consciousness of personal risk and sacrifice. Is it in these circumstances a reasonable and fair thing to leave the choice, aye or no, purely to the judgment of the individual practitioner? If this is done it is obvious there can be no common and impartial standard, and inevitably there will be great inequality of sacrifice. The more sensitive will suffer out of proportion to their fellows, and there is even the risk that in the absence of direction and guidance there may be so much delay in decision that the response will not be equal to the demand. But if the issue ought not to be left entirely to personal debate there is only one other possible course—namely, to substitute for the individual judgment a corporate or professional decision. The more clearly it is recognised that the practitioners concerned are under the pressure of

W1 HO 6AX

conflicting claims as these are defined respectively by personal obligations and by patriotic motives, the more obvious does it appear that the only method of relief is the interposition of a competent, a sympathetic, and an impartial tribunal. In a word, the profession must take action as a whole and must not leave the painful and anxious decision to the judgment of its perplexed and unguided members.

The plan for organisation and for corporate action in this matter presents a claim for sacrifice from many individuals who will, as a matter of fact, never be called upon to leave home for active service. The sacrifice is a willingness to leave the definition of their personal duty in the hands of others. Undoubtedly this to men acutely conscious of many personal claims and obligations is a hard thing to yield. But what is the alternative? It is that there shall be no organisation at all, and that to every man it shall be left to do what seems right in his own eyes. Between the two courses there is no middle way. The Army needs more doctors. More doctors, therefore, must certainly be found. Who shall go and who stay are [illegible] tions of great difficulty and stress to the [illegible] constituency as it at present exists. Un[illegible] choice with all its embarrassments is to be [illegible] the individual, the decision must be refe[illegible] some authoritative voice. The Central W[illegible] mittee, whatever its demerits, is at least an organisation which is trying to arrange a decision based upon an appreciation of the whole of the facts. Still more, it is in active existence. In the absence of competitors it holds the field, and this in the existing situation is no small virtue. Hence, though we have criticised some of its arrangements, we hold that it deserves the support of the profession, and that in the present circumstances it is the only available medium through which can be secured, with some measure of justice and of equality of sacrifice, the professional assistance of which, now admittedly, the Army stands in need. Not less essential is the Committee in order to maintain such a distribution of the profession that the needs of the civil population may be reasonably satisfied.

